# Supplementary material for: Circulating Extracellular Vesicles Impair Mesenchymal Stromal Cell Differentiation Favoring Adipogenic Rather than Osteogenic Differentiation in Adolescents with Obesity
Source: Int J Mol Sci. 2022 Dec 27;24(1):447. doi: 10.3390/ijms24010447 (PMC9820591; doi:10.3390/ijms24010447)
Supplement: Supplementary file 1 [file ijms-24-00447-s001.zip › Supplementary Figures.pptx]

## Slide 1
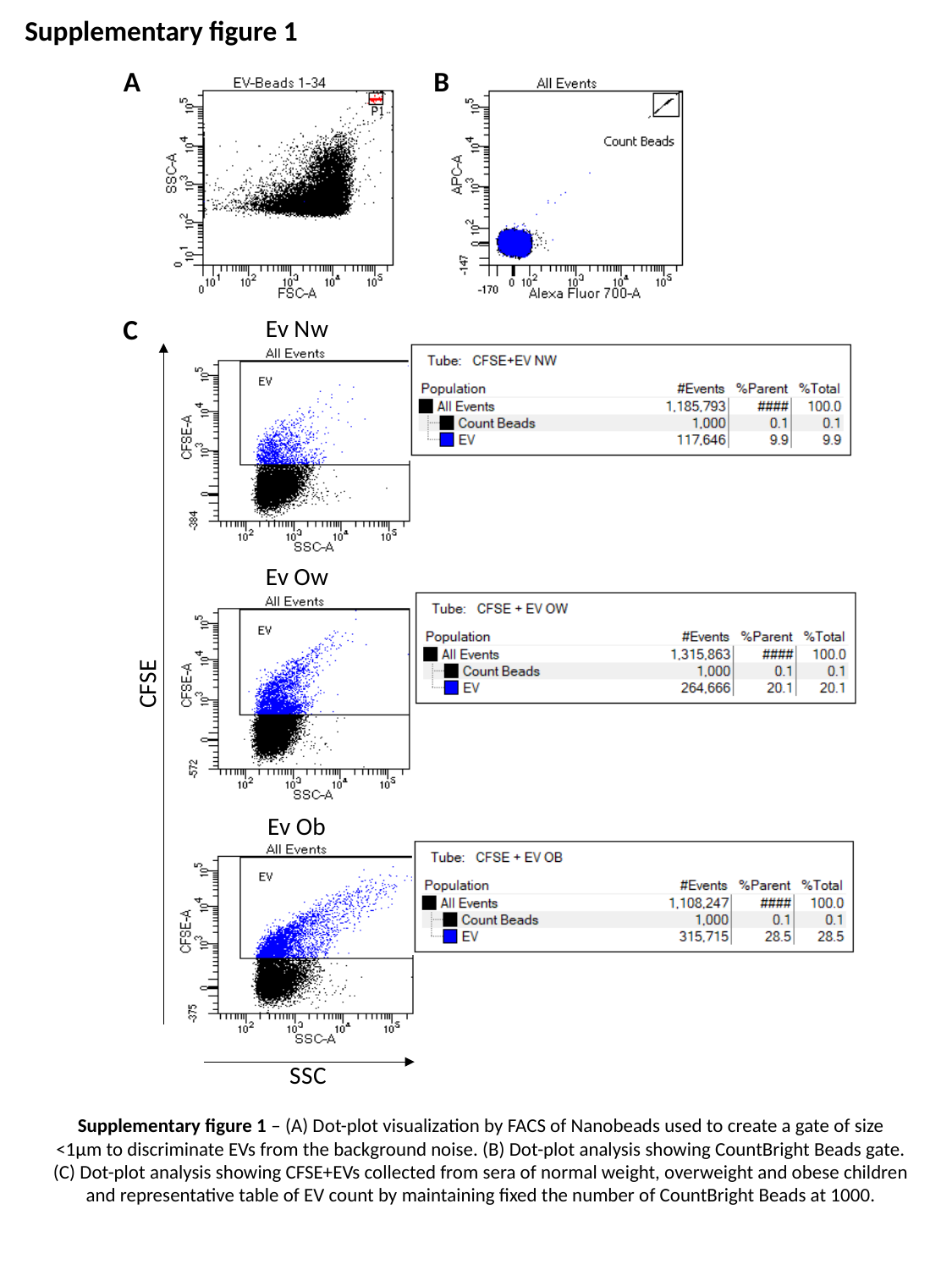

Supplementary figure 1
Supplementary figure 1 – (A) Dot-plot visualization by FACS of Nanobeads used to create a gate of size <1μm to discriminate EVs from the background noise. (B) Dot-plot analysis showing CountBright Beads gate. (C) Dot-plot analysis showing CFSE+EVs collected from sera of normal weight, overweight and obese children and representative table of EV count by maintaining fixed the number of CountBright Beads at 1000.

## Slide 2
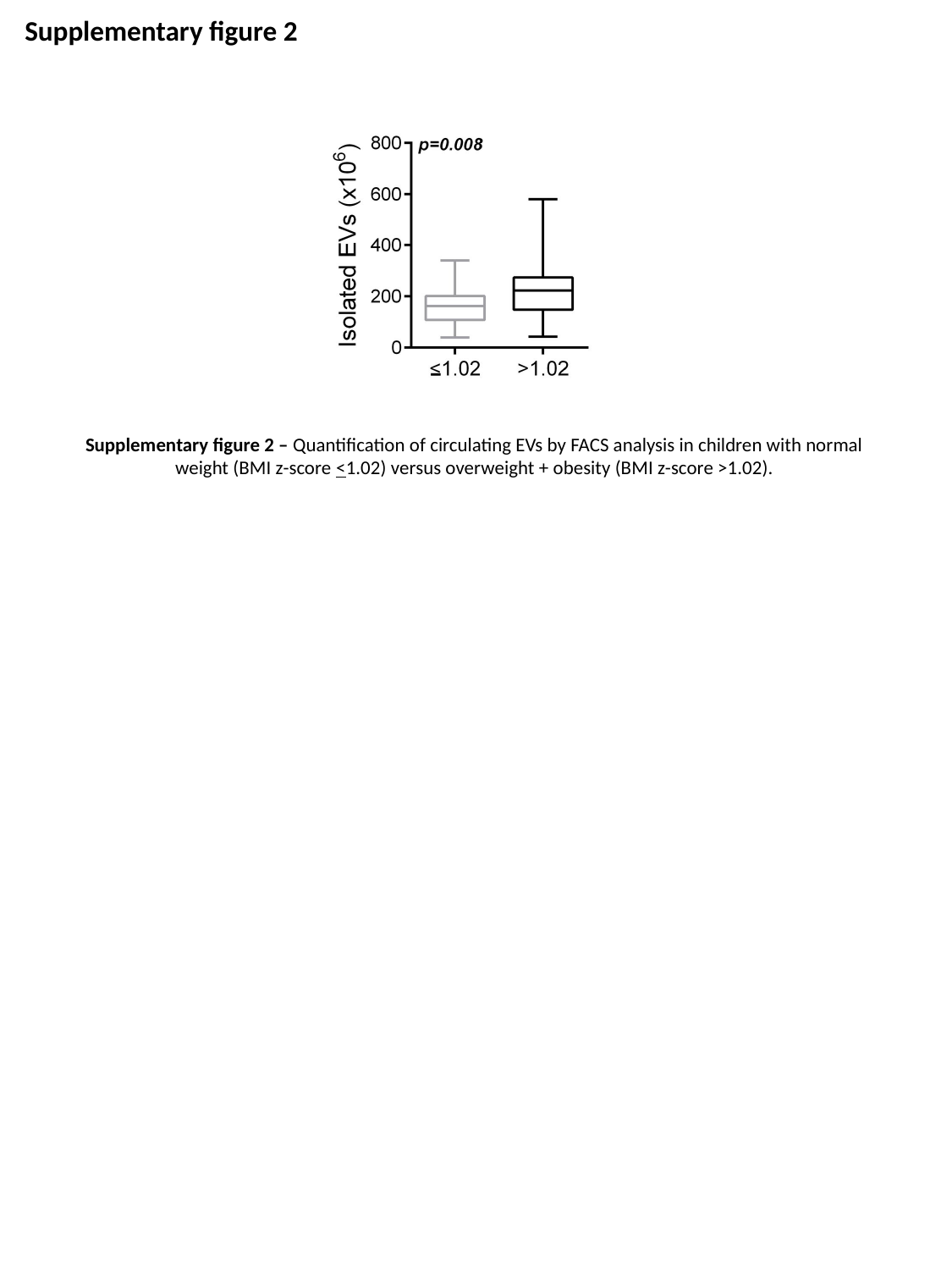

Supplementary figure 2
Supplementary figure 2 – Quantification of circulating EVs by FACS analysis in children with normal weight (BMI z-score <1.02) versus overweight + obesity (BMI z-score >1.02).

## Slide 3
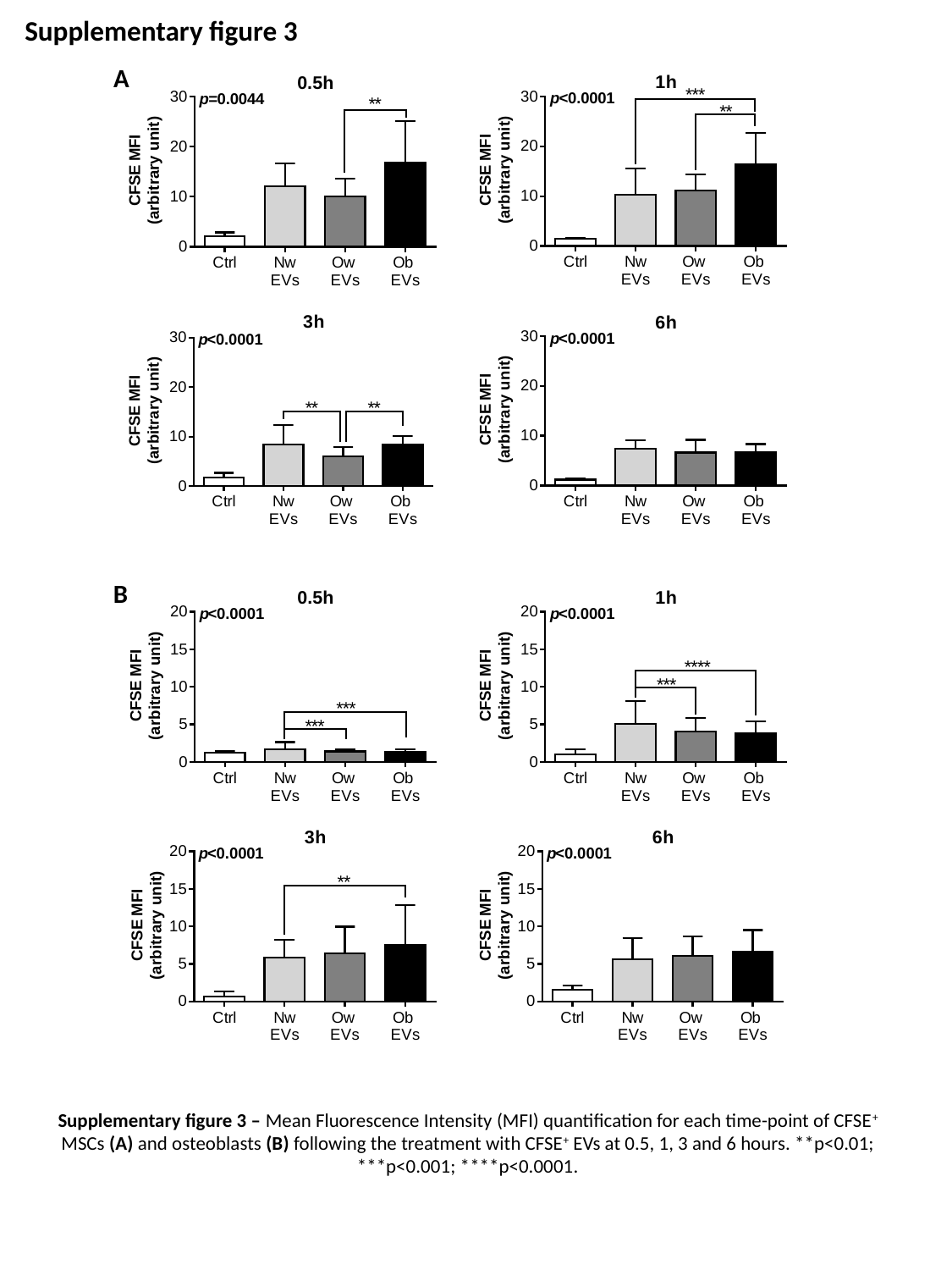

Supplementary figure 3
Supplementary figure 3 – Mean Fluorescence Intensity (MFI) quantification for each time-point of CFSE+ MSCs (A) and osteoblasts (B) following the treatment with CFSE+ EVs at 0.5, 1, 3 and 6 hours. **p<0.01; ***p<0.001; ****p<0.0001.

## Slide 4
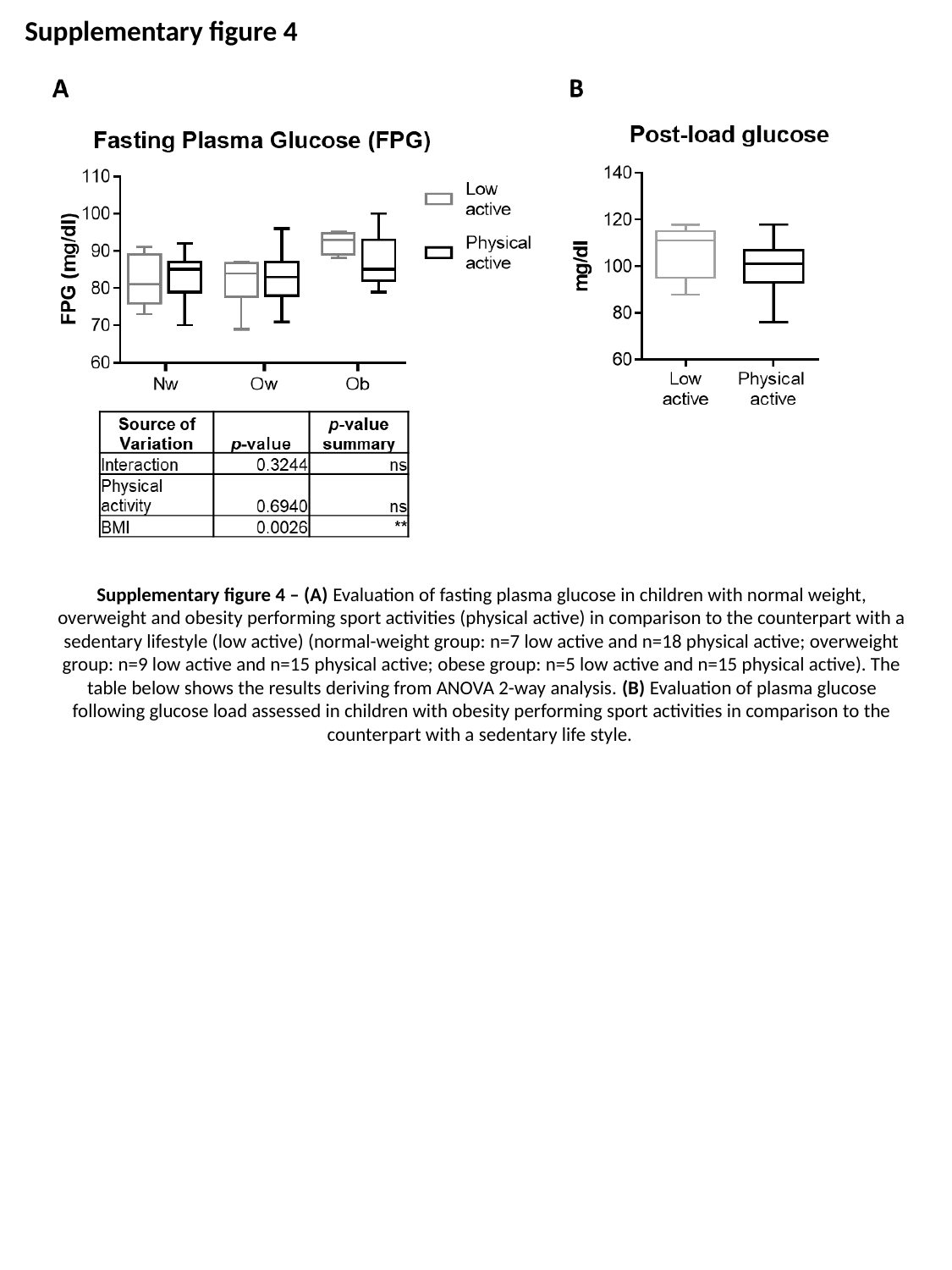

Supplementary figure 4
Supplementary figure 4 – (A) Evaluation of fasting plasma glucose in children with normal weight, overweight and obesity performing sport activities (physical active) in comparison to the counterpart with a sedentary lifestyle (low active) (normal-weight group: n=7 low active and n=18 physical active; overweight group: n=9 low active and n=15 physical active; obese group: n=5 low active and n=15 physical active). The table below shows the results deriving from ANOVA 2-way analysis. (B) Evaluation of plasma glucose following glucose load assessed in children with obesity performing sport activities in comparison to the counterpart with a sedentary life style.
